# Dyslipidemia and Food Security in Low-Income US Adolescents: National Health and Nutrition Examination Survey, 2003–2010

**DOI:** 10.5888/pcd13.150441

**Published:** 2016-02-11

**Authors:** June M. Tester, Barbara A. Laraia, Cindy W. Leung, Michele L. Mietus-Snyder

**Affiliations:** Author Affiliations: Barbara A. Laraia, University of California, Berkeley, School of Public Health, Berkeley, California; Cindy W. Leung, University of California, San Francisco, Center for Health and Community, San Francisco, California; Michele L. Mietus-Snyder, Center for Translational Science, Children’s National Health System, Washington, DC.

## Abstract

**Introduction:**

Low levels of food security are associated with dyslipidemia and chronic disease in adults, particularly in women. There is a gap in knowledge about the relationship between food security among youth and dyslipidemia and chronic disease. We investigated the relationship between food security status and dyslipidemia among low-income adolescents.

**Methods:**

We analyzed data from adolescents aged 12 to 18 years (N = 1,072) from households with incomes at or below 200% of the federal poverty level from the National Health and Nutrition Examination Survey (NHANES) 2003–2010. We used logistic regression to examine the relationship between household food security status and the odds of having abnormalities with fasting total cholesterol (TC), low-density lipoprotein cholesterol (LDL-C), serum triglycerides (TGs), high-density lipoprotein cholesterol (HDL-C), TG/HDL-C ratio, and apolipoprotein B (Apo B). Models included age, sex, race/ethnicity, smoking status, partnered status in the household, and maternal education, with additional adjustment for adiposity.

**Results:**

Household food security status was not associated with elevated TC or LDL-C. Adolescents with marginal food security were more likely than food-secure peers to have elevated TGs (odds ratio [OR] = 1.86; 95% confidence interval [CI], 1.14–3.05), TG/HDL-C ratio (OR = 1.74; 95% CI, 1.11–2.82), and Apo B (OR = 1.98; 95% CI, 1.17–3.36). Female adolescents with marginal food security had greater odds than male adolescents of having low HDL-C (OR = 2.69; 95% CI, 1.14–6.37). No elevated odds of dyslipidemia were found for adolescents with low or very low food security. Adjustment for adiposity did not attenuate estimates.

**Conclusion:**

In this nationally representative sample, low-income adolescents living in households with marginal food security had increased odds of having a pattern consistent with atherogenic dyslipidemia, which represents a cardiometabolic burden above their risk from adiposity alone.

## Introduction

Food security is defined as having “consistent, dependable access to enough food for active, healthy living” (1). From 2003 through 2005, national data showed that 11.6% of households had low or very low food security, and an additional 8.1% had marginal food security (2). In the past decade interest has increased in understanding the ways in which living with low levels of food security affects chronic disease risk (3–6). Although evidence points toward glycemic control and high blood pressure in particular (3–6), the link between food security and dyslipidemia is less clear (3,4,7,8).

One study of adults in the National Health and Nutrition Examination Survey (NHANES 1999–2004) showed that although adults with low and very low food security (often combined and referred to as “food insecurity”) (1) more frequently reported that they had “high blood cholesterol” than did their food-secure coutnerparts, there was no association with laboratory evidence of having elevated total cholesterol (TC) or elevated low-density lipoprotein cholesterol (LDL-C) (3). A separate analysis in NHANES (2003–2008) evaluated 10-year cardiovascular disease risk, which is calculated by an algorithm that incorporates risk factors such as age and blood pressure, along with 2 dyslipidemia measures (TC and high-density lipoprotein cholesterol (HDL-C), adjusted for race and ethnicity, known to modulate lipid metabolism. Although adults with very low food security had a higher 10-year risk than did adults with full food security, there were no individual associations with having elevated TC or HDL‑C (4). However, other investigators found evidence suggesting that the relationship between food security and dyslipidemia is different for men than for women. An analysis of adults in NHANES (1999–2002) reported higher odds of elevated serum triglycerides (TGs) among food-insecure women and higher LDL-C and TG/HDL-C ratio among marginally food-secure women, compared with their male counterparts (7). Finally, a recent study of adults in Wisconsin assessed food security with a modified 2-item tool and found higher odds of low HDL-C among food-insecure women but not among food insecure men (8).

Thus far, no studies to our knowledge have evaluated potential associations between dyslipidemia and food insecurity in children or adolescents. This lack of research is an important gap. There is a long natural history for the progression of cardiovascular disease and atherosclerosis; build-up of lipid deposit in the inner layer of arteries produces fatty streaks and begins in early childhood (9). Like adults, food-insecure adolescents report higher consumption of high-fat fast foods (10) than their food-secure peers; food-insecure youth also have a higher intake of refined carbohydrates, and added sugars (10,11). Using a nationally representative sample of low-income adolescents, we examined whether lower levels of food security contribute to a greater risk of dyslipidemia in adolescents compared with peers with high food security and tested whether an individual’s sex influenced the relationship between food security and dyslipidemia.

## Methods

### Study population

We examined data from participants aged 12 to 18 years from NHANES, which uses a complex, multistage probability sample designed to be representative of the US civilian noninstitutionalized population. Data included responses to questionnaires and the results of physical examinations and laboratory tests conducted in Mobile Examination Centers (MEC). Our analysis combined data from 2003 through 2010.

There were 2,453 adolescents in the fasting subsample. Because upper-income households are unlikely to experience food insecurity, we restricted the analysis to data on low-income households, with the cut point of being at or below 200% of the federal poverty level (FPL) (3), reducing the available sample to 1,326. Adolescents were excluded if they were pregnant or missing anthropometric data, food security data, lipid panel, or any of the covariates, yielding a final sample of 1,072 adolescents.

### Measures

Race/ethnicity was self-reported by survey participants, and household income was reported by an adult household representative. Annual household income and family size are used with each cycle of NHANES to calculate the FPL in accordance with the poverty guidelines of the US Department of Health and Human Services’ (12).

Household food security status was assessed from an adult caregiver (the household representative) using the 18-item Core Food Security Module, a well-validated, staged questionnaire in English and in Spanish developed by the US Department of Agriculture (USDA) to measure food security during the previous 12 months (13). Questions are asked about such issues as anxiety about running out of food, relying on a few low-cost foods to feed the children because of lack of money, and curbing meals because of lack of money. Households with no affirmative answers were categorized as having high food security, those with 1 or 2 affirmative answers as having marginal food security, those with 3 to 7 affirmative answers as having low food security, and those with 8 to 18 affirmative answers as having very low food security, using revised language for food security categories defined by the USDA in 2006 (14).

Height and weight were measured by trained personnel at the mobile examination centers using stardardized protocols (15). Adolescents aged 12 years or older were eligible to participate in the fasting laboratory subsample. Lipid panel data included in these analyses were TC; HDL-C; and calculated LDL-C, TG, and apolipoprotein B (Apo B) (15). 

Lipid levels are known to vary with age; there can be as much as a 20% decrease in TC and LDL-C during adolescence (16). Because of these changes during adolescence, Joliffe and Janssen developed age- and sex-specific cutoffs for lipoprotein levels derived from cross-sectional data on adolescents in NHANES (17). Subsequent testing showed that these NHANES cutoffs most accurately predict future abnormal adult dyslipidemia for HDL-C but that National Cholesterol Education Program (NCEP) cut points for TC, LDL-C, and TG levels in adolescence were still preferable for prediction of later dyslipidemia in adulthood (18). We used the published age- and sex-specific NHANES cutoffs for low HDL-C (range: 39.8–43.7 mg/dL for boys and 39.8–40.2 mg/dL for girls, aged 12 to 18 years) (17,18) and used the NCEP cutoffs for borderline elevation (TGs ≥90 mg/dL, TC ≥170 mg/dL, and LDL-C ≥110 mg/dL), which correspond to roughly the 75th percentile for children aged 2 to 19 years (18,19).

TG/HDL-C ratio is a well-known predictor of cardiovascular disease in adults, and having a TG/HDL-C ratio above 2.0 is linked to clinically meaningful cardiovascular disease surrogates in children (20,21). We used Apo B level because of its value as a predictor of the total LDL-C particle burden, because every non-HDL-C particle contains a single signature Apo B molecule. Apo B was categorized as elevated if at or above 90 mg/dL, which corresponds roughly to the 75th percentile for this age group (22).

We adjusted for the relationship between food insecurity and each clinical marker of dyslipidemia with age (in years), sex, race/ethnicity (Non-Hispanic white, Hispanic, Non-Hispanic black, other), poverty income ratio (0%–50%, 50.1%–100%, 100.1%–150%, and 150.1%–200% of the FPL), married/partnered status in the household, and maternal education. Maternal education was derived from whether the female household respondent (or the spouse of the respondent if male) was a high school graduate. Because of missing data about reported smoking, we used serum cotinine levels to define an active smoker (>15 ng/ml) versus a nonsmoker (≤15 ng/ml) (23).

### Statistical analysis

Sample weights were used to account for the complex, multistage, probability sampling design used in NHANES during the 8-year period (2003–2010). Because our analyses of lipid outcomes were limited to the roughly half of adolescents who were in the fasting subsample, fasting subsample weights were used to account for the additional stage of sampling and for nonresponse.

We compared characteristics of the sample using unadjusted linear regression of continuous variables (eg, age) across food security taken as a categorical variable. An adjusted Wald test was done as a postestimation to calculate the F statistic. For categorical variables (eg, race/ethnicity), we performed χ^2^ tests using a design-based F statistic.

We conducted logistic regression of the odds of abnormal levels of each of the respective lipid outcomes with household food security taken as a 4-category variable (food secure, marginal food security, low food security, and very low food security), adjusting for covariates. Because of previous literature suggesting that the association between food security and dyslipidemia may vary by sex, we tested for interactions between food insecurity and sex for each laboratory outcome. An adjusted Wald test was performed using *P* < .15 as a cutoff for significant effect modification.

In addition to these covariates, we added a model that adjusted for adiposity, because adiposity may be on the causal pathway in the relationship between our main variables of interest and dyslipidemia. Body mass index (BMI) and waist-to-height ratio were both considered. Waist-to-height ratio is a useful measure of visceral adiposity that is increasingly recognized as being more closely associated with cardiometabolic health than BMI (24). Because waist-to-height ratio was more consistently associated with dyslipidemia outcomes than was BMI in both univariate and multivariate models and also because models with waist-to-height ratio influenced estimates more than did inclusion of BMI, models adjusted with waist-to-height ratio are shown. Waist-to-height ratio is considered elevated when it is above 0.5 (24). Waist-to-height ratio was scaled (20×) for more meaningful interpretation of the odds associated with incremental increases of 0.05 in waist-to-height ratio .

All analyses were conducted using Stata 12.1 (StataCorp LP). This study did not require insititutional review board approval, because it was a secondary data analysis and did not include personally identifying information; it was, therefore, determined not to be human subjects research.

## Results

Survey-weighted proportions of participants with high, marginal, low, and very low food security were 53%, 12%, 22%, and 13%, respectively (Table 1). Age of participants was not equivalent between these groups; mean age was lower in the groups with lower food security. There were no differences in mean BMI or waist-to-height ratio by age. There were no significant differences among low-income adolescents by food security status in terms of income and maternal education. However, significant differences were found by food security status with respect to race/ethnicity (Hispanic and black participants), partnered status of the household, sex, and whether the adolescent was a smoker.

**Table 1:** Demographic Characteristics of Adolescents Aged 12–18 Years (N = 1,072),^a^^,^^b^ by Food Security Status, National Health and Nutrition Examination Survey, 2003–2010

Characteristic	Food Security (N = 512)	Marginal Food Security (N = 152)	Low Food Security (N = 260)	Very Low Food Security (N = 148)	*P *Value^c^
**Mean age, y (SD)**	15.0 (14.9–15.1)	14.7 (14.5–14.9)	14.3 (14.1–14.5)	14.8 (14.6–15.0)	.002
**Mean BMI, kg/m^2^ (SD)**	23.2 (22.9–23.5)	23.9 (23.3–24.5)	23.6 (23.2–24.0)	23.9 (23.2–24.6)	.94
**Waist-height ratio, mean (SD)**	0.489 (0.484–0.494)	0.497 (0.488–0.506)	0.495 (0.488–0.502)	0.500 (0.490–0.510)	.92
**Sex, no. (%)**					
Male	276 (53.0)	61 (36.3)	142 (52.8)	79 (48.2)	.05
Female	236 (47.0)	91 (63.7)	118 (47.2)	69 (51.8)	
**Race/ethnicity, no. (%)**					
Non-Hispanic white	113 (49.8)	17 (30.4)	30 (30.6)	34 (46.7)	.004
Non-Hispanic black	140 (16.1)	56 (29.9)	91(29.7)	50 (18.7)	
Hispanic	227 (24.0)	73 (35.6)	133 (34.4)	59 (31.3)	
Other/mixed	32 (10.1)	6 (4.1)	6 (5.2)	5 (3.3)	
**FPL, no. (%)**					
0–50	81 (13.0)	28 (13.6)	66 (23.2)	33 (19.0)	.18
50.1–100	143 (27.5)	48 (29.1)	93 (30.0)	56 (37.4)	
100.1–150	159 (30.6)	45 (31.3)	66 (28.7)	40 (31.5)	
150.1–200	129 (28.9)	31 (26.0)	35 (18.1)	19 (12.1)	
**Marital status, no. (%)**					
Single/divorced	193 (34.0)	66 (43.8)	121 (48.6)	82 (52.4)	.01
Married/partnered	319 (66.0)	86 (56.2)	139 (51.4)	66 (47.6)	
**Education level, no. (%)**					
Less than high school	224 (30.5)	62 (33.4)	139 (44.6)	65 (40.1)	.10
≥High school graduate	288 (69.5)	90 (66.6)	121 (55.4)	83 (59.9)	
**Smoking status,^d^ no. (%)**					
Nonsmoker	472 (91.2)	140 (90.0)	237 (88.8)	123 (77.4)	.02
Smoker	40 (8.8)	12 (9.1)	23 (11.2)	25 (22.6)	

Abbreviations: BMI, body mass index; FPL, federal poverty level; SD, standard deviation.

a All were from the fasting subsample and were low-income (FPL ≤200%). All data were weighted.

b Household food security status was assessed using a validated 18-item instrument; an adult caregiver was asked about anxiety about running out of food, relying on a few low-cost foods to feed the children because of lack of money, and curbing meals because of lack of money. Households with no affirmative answers were categorized as having high food security, those with 1 or 2 affirmative answers as having marginal food security, those with 3 to 7 affirmative answers as having low food security, and those with 8 to 18 affirmative answers as having very low food security.

c *P* values for age, BMI, and waist-height ratio derived from F statistic from postestimation Wald test after unadjusted regression between continuous outcome variable and categorical food security; for all other variables, *P* values were derived from χ^2^ test, using design-based F statistic.

d Serum cotinine levels measured to determine smoking status: >15 ng/ml = smoker; ≤15 ng/ml = nonsmoker.

Although there were no significant differences among adolescents with respective dyslipidemia outcomes, there was a trend toward worse lipid profiles (with the exception of TC and LDL-C) for adolescents in marginally food secure households compared with their peers (Table 2). Odds of having either TC of 170 mg/dL or higher or LDL-C of 110 mg/dL or higher were not associated with food security status (Table 3). Odds of having elevated TG (≥90 mg/dL) were significantly associated with marginal food security (odds ratio [OR] = 1.86; 95% confidence interval [CI], 1.14–3.05) but not with low or very low food security. Odds of having an elevated TG/HDL-C ratio were significantly associated with marginal food security (OR = 1.74; 95% CI, 1.09–2.78) but not with low or very low food security. Odds of having an Apo B level at or above 90 mg/dL were associated with marginal food security (OR = 1.98; 95% CI, 1.17–3.36) but not with low or very low food security.

**Table 2:** Proportion of Adolescents Aged 12–18 Years With Dyslipidemia (N = 1,072),^a^ by Food Security Status, National Health and Nutrition Examination Survey, 2003–2010^a^

Characteristic	Food Security (N = 512)	Marginal Food Security (N = 152)	Low Food Security (N = 260)	Very Low Food Security (N = 148)	*P *Value^b^
	No. (%)				
High TC (≥170 mg/dL)	162 (32.7)	45 (29.0)	71 (26.6)	45 (26.5)	.46
High LDL-C (≥110 mg/dL)	89 (19.3)	28 (18.6)	40 (17.5)	26 (17.1)	.93
High TG (≥90 mg/dL)	145 (29.5)	46 (37.4)	79 (35.7)	53 (35.9)	.40
Low HDL-C NHANES (≤40 mg/dL)	72 (14.7)	20 (18.0)	34 (14.5)	18 (13.0)	.76
High TG/HDL-C ratio (≥2.0)	130 (28.6)	39 (34.0)	62 (28.8)	45 (31.5)	.76
High Apo B (≥90 mg/dL)	50 (15.3)	21 (22.8)	30 (15.0)	14 (14.3)	.35

Abbreviations: Apo B, apolipoprotein B; HDL-C, high-density lipoprotein cholesterol; LDL-C, low-density lipoprotein cholesterol; TC, total cholesterol; TG, triglycerides.

a Household food security status was assessed using a validated 18-item instrument; an adult caregiver was asked about anxiety about running out of food, relying on a few low-cost foods to feed the children because of lack of money, and curbing meals because of lack of money. Households with no affirmative answers were categorized as having high food security, those with 1 or 2 affirmative answers as having marginal food security, those with 3 to 7 affirmative answers as having low food security, and those with 8 to 18 affirmative answers as having very low food security.

b *P* values derived from χ^2^ test, using design-based F test. All data were weighted.

**Table 3:** Odds of Dyslipidemia in Low-Income Adolescents, by Food Security Status, National Health and Nutrition Examination Survey 2003–2010 **a ^,^ b**

Characteristic	Model 1 (N = 1,072)			Model 2 (N = 1,049)		
	Marginal Food Security	Low Food Security	Very Low Food Security	Marginal Food Security	Low Food Security	Very Low Food Security
	Odds Ratio (95% Confidence Interval)					
**TC (≥ 170 mg/dL)**	0.82 (0.52–1.29)	0.80 (0.46–1.37)	0.80 (0.48–1.33)	0.78 (0.49–1.26)	0.80 (0.47–1.36)	0.79 (0.46–1.34)
**LDL-C (≥ 110 mg/dL)**	0.97 (0.57–1.66)	0.97 (0.53–1.78)	0.90 (0.48–1.69)	0.90 (0.50–1.56)	0.74 (0.39–1.41)	0.86 (0.44–1.71)
**TG (≥90 mg/dL)**	1.86 (1.14–3.05)	1.57 (0.94–2.62)	1.31 (0.80–2.15)	1.84 (1.07–3.16)	1.56 (0.91–2.67)	1.21 (0.73–2.02)
**TG/HDL-C ratio (≥2.0)**	1.74 (1.09–2.78)	1.15 (0.71–1.88)	1.05 (0.63–1.77)	1.81 (1.05–3.10)	1.13 (0.65–1.97)	0.95 (0.54–1.67)
**Apo B (≥90 mg/dL)**	1.98 (1.17–3.36)	1.14 (0.56–2.34)	0.97 (0.48–1.97)	2.13 (1.20–3.79)	1.12 (0.50–2.48)	0.82 (0.37–1.82)
**Low HDL-C (**≤**40 mg/dL)^c^**						
Female	2.69 (1.14–6.37)	1.19 (0.44–3.20)	0.51 (0.44–3.20)	2.94 (1.17–7.40)	1.14 (0.39–3.32)	0.54 (0.14–2.12)
Male	0.84 (0.28–2.52)	0.95 (0.48–1.86)	0.84 (0.34–2.10)	1.0 (0.28–3.60)	0.97 (0.44–2.12)	0.69 (0.25–1.91)

Abbreviations: Abbreviations: Apo B, apolipoprotein B; HDL-C, high-density lipoprotein cholesterol; LDL-C, low-density lipoprotein cholesterol; TC, total cholesterol; TG, triglycerides.

a Model 1 includes age, sex, race/ethnicity, marital status, and maternal education; model 2 adds additional adjustment for adiposity using waist-to-height ratio. Food-secure adolescents is the reference group.

b Household food security status was assessed using a validated 18-item instrument; an adult caregiver was asked about anxiety about running out of food, relying on a few low-cost foods to feed the children because of lack of money, and curbing meals because of lack of money. Households with no affirmative answers were categorized as having high food security, those with 1 or 2 affirmative answers as having marginal food security, those with 3 to 7 affirmative answers as having low food security, and those with 8 to 18 affirmative answers as having very low food security.

c Results for odds of low HDL-C are stratified by sex because of a significant interaction between sex and food security.

The interaction between food security and sex was significant for HDL-C (*P* =.14), and sex-stratified results are therefore presented. In female adolescents, odds of low HDL-C were associated with marginal food security (OR = 2.69; 95% CI, 1.14–6.37), although not with low or very low food security. No associations were seen in male adolescents.

With the exception of TC, waist-to-height ratio was independently associated with every dyslipidemia outcome at the level of *P* < .05. Waist-to-height ratio ranged between 0.49 (for food-secure adolescents) and 0.50 (for adolescents with very low food security) (Table 1), and adjusted ORs for abnormal LDL-C, TGs, HDL-C, TG/HDL-C ratio, and Apo B were 1.17, 1.40, 1.62, 1.57, and 1.43, respectively. These findings mean that every 0.05 increase in waist-to-height ratio was associated with a 17% to 62% increase in odds of dyslipidemia. However, addition of waist-to-height ratio to the models did not attenuate the estimates of odds of dyslipidemia associated with varying food security status (Table 3), suggesting that the relationships seen for adolescents with marginal food security did not appear to be mediated by adiposity.

## Discussion

The findings from this representative sample suggest that the experience of marginal household food security is associated with the pattern of atherogenic dyslipidemia in US adolescents rather than with elevated cholesterol. Elevated TC or LDL-C would have been a plausible finding, because food-insecure adolescents reportedly consume more fast foods than do their food-secure coutnerparts (10), and consumption of foods high in saturated fats leads to increases in LDL-C and TC (25). However, instead of an elevation in TC or LDL-C, these adolescents showed higher odds of having an elevation in TGs, which is consistently seen in individuals with diets that have a high proportion of calories coming from sugars such as sucrose and fructose (26). It may be that, among these adolescents, consumption is disproportionately tipped toward calories from added sugars and refined carbohydrates (eg, sugary beverages) than toward foods rich in saturated fats (eg, red meat).

Findinga on marginally food-secure adolescents were consistent with the “atherogenic triad,” which is characterized by elevated TGs, low HDL-C, and a preponderance of small, dense LDL-C particles (27). In atherogenic dyslipidemia, a high burden of small, dense LDL-C particles contributes to cardiovascular disease risk despite normal (or only minimally elevated) levels of LDL-C. This is attributed to decreased clearance of small LDL-C and therefore increased circulation time and greater infiltration and inflammation in the arterial intima (27). The pattern of atherogenic dyslipidemia is strongly associated with visceral adiposity, and yet adjustment with waist-to-height ratio in our model did not attenuate estimates of odds of dyslipidemia outcomes. This finding suggests that these adolescents in marginally food secure households may have a cardiovascular health burden that is above the risk conferred by their adiposity alone.

Adult data indicate that inflammation is a potential mediator of the association between food insecurity and a 20% increased risk of cardiovascular disease (4). Inflammation triggers insulin resistance during perceived stress, in an effort to keep glucose available to meet the metabolic needs of an activated and energy-intensive immune system. This fact helps to explain the shift of energy toward protein catabolism and gluconeogenesis in insulin resistance, combined dyslipidemia, and diabetes (28). Unfortunately, the insulin resistant reallocation of resources persists in chronic stress conditions such as food insecurity and may be aggravated by stress eating, accentuating risk for dyslipidemia. More research is needed to characterize potential interactions between stress, dietary intake, and dyslipidemia in adolescents.

It is not clear why this elevated risk was seen in marginally food-secure adolescents but not in their counterparts with lower levels of food security; however, this finding is consistent with finding in the existing literature that the experience of individuals with marginal food security is distinct from those with lower levels of food security (2). It may be that marginal food security in adolescents is associated with differential dietary intake (eg, disproportionate discretionary calories). In particular, increased consumption of added sugar is a contributor to atherogenic dyslipidemia, increasing TGs and cardiovascular disease risk (26); more research is needed to clarify differences in the dietary intake of adolescents with marginal food security compared with their peers. Indeed, sex-stratified data from NHANES (which uses the standard USDA Core Food Security Module [13]) showed differing relationships between dyslipidemia outcomes in marginally food secure compared with fully food insecure individuals (7). Furthermore, the analysis of Wisconsin adults that found a relationship with low HDL-C in women used an alternative definition for food insecurity that may capture less severe food insecurity and marginal food security along with more severe food insecurity (8).

The findings of this analysis support previously reported findings that suggest that the relationship between food security and low HDL-C may be moderated by sex (8). The varying experience of household food insecurity between males and females may contribute to these observed differences. Cortisol excretion is associated with lower HDL-C (29), and a disproportionate stress response among females could contribute to a disproportionate lowering of HDL-C. Additionally, depression is more common in women (30) and is associated with lower HDL-C (31). It is possible that marginal food security and food insecurity are associated with higher depressive symptoms among female adolescents. 

Participation in the Supplemental Nutrition Assistance Program (SNAP, or “food stamps”) is a complex consideration for any analysis that considers food insecurity. Because of underlying issues with self-selection and endogeneity, some researchers cite fundamental concerns with modeling SNAP participation and food security simultaneously when using observational data rather than experimental methods (32). For this reason, as food insecurity was our main predictor of interest, we omitted SNAP status. Further analysis is required to understand whether increased access to public resources such as SNAP may attenuate the physiologic impact of food insecurity in the most severely affected families.

Our study has strengths, one of which is the use of nationally representative data. Furthermore, this analysis considers adiposity using a measure that is more aligned with cardiometabolic risk than is BMI. Our study also has limitations. As with any cross-sectional analysis, causal interpretation of findings is limited. There were covariates that could not be included, such as adolescents’ physical activity levels, and their omission introduces residual confounding. Respondents rated their household food security in the previous 12 months, which is a long period during which changes in diet could influence the level of dyslipidemia. Furthermore, a more thorough consideration of atherogenic dyslipidemia would have included a direct examination of LDL-C particle number and LDL-C particle size, which was not collected by NHANES.

In summary, this analysis of nationally representative data show that adolescents with marginal food security had a pattern consistent with atherogenic dyslipidemia. Compared with peers in food secure households, they had greater odds of having elevated TGs, elevated Apo B, and an elevated TG/HDL-C ratio. Adjustment for waist-to-height ratio changed estimates only minimally. In particular, female adolescents from margially food secure households had nearly a threefold increase in odds of having low HDL-C than did their male counterparts. Taken together, these findings suggest that disproportionate cardiovascular disease burden among adolescents with marginal food security is above the risk from adiposity alone.
